# Multilevel Evolutionary Algorithm that Optimizes the Structure of Scale-Free Networks for the Promotion of Cooperation in the Prisoner’s Dilemma game

**DOI:** 10.1038/s41598-017-04010-2

**Published:** 2017-06-28

**Authors:** Penghui Liu, Jing Liu

**Affiliations:** Key Laboratory of Intelligent Perception and Image Understanding of Ministry of Education, Xidian University, Xi’an, 710071 China

## Abstract

Understanding the emergence of cooperation has long been a challenge across disciplines. Even if network reciprocity reflected the importance of population structure in promoting cooperation, it remains an open question how population structures can be optimized, thereby enhancing cooperation. In this paper, we attempt to apply the evolutionary algorithm (EA) to solve this highly complex problem. However, as it is hard to evaluate the fitness (cooperation level) of population structures, simply employing the canonical evolutionary algorithm (EA) may fail in optimization. Thus, we propose a new EA variant named mlEA-C_PD_-SFN to promote the cooperation level of scale-free networks (SFNs) in the Prisoner’s Dilemma Game (PDG). Meanwhile, to verify the preceding conclusions may not be applied to this problem, we also provide the optimization results of the comparative experiment (EA_cluster_), which optimizes the clustering coefficient of structures. Even if preceding research concluded that highly clustered scale-free networks enhance cooperation, we find EA_cluster_ does not perform desirably, while mlEA-C_PD_-SFN performs efficiently in different optimization environments. We hope that mlEA-C_PD_-SFN may help promote the structure of species in nature and that more general properties that enhance cooperation can be learned from the output structures.

## Introduction

The Prisoner’s Dilemma Game (PDG) is a popular abstract mathematical method and has been employed in biology to explain the emergence and persistence of cooperation behavior among selfish individuals^1–8^. After all, survival of the fittest is a widely accepted natural selection rule, and individuals employing the selfish strategy might be expected to be more likely to persist. After carefully studying PDG, researchers found that organisms may still form a cooperative community even if they all act entirely for their own interest. Even so, researchers still found it hard to explain large-scale cooperation in reality, as defection usually dominates in their simulations. To explain this puzzle, researchers have long been exploiting the deeper mechanisms.

In the past decades, network reciprocity, proposed by Nowak *et al*., has had wide influence in this avenue of research. Individuals are constrained by spatial structure to play only with their immediate neighbors^8^. Nowak *et al*. concluded that topology constraints influence the evolution of cooperation (confirmed years later). After that, many extended studies have contributed to network reciprocity. In the early stages, researchers focused on the single layer networks: They found that population structure plays a determinate role in the evolution of cooperation^9^ and cooperators in PDG are likely to form clusters to defend against defectors^10^. They revealed the potential positive relationship between cooperation and some network properties, such as heterogeneity^7, 11^ and clustering coefficients^12^. And they also focused on how error and attack on the poulation structures may influence the evolution of cooperation^13^. Recently, researchers have analyzed the evolutionary game in interdependent networks, as populations in reality are not isolated and interaction exists between different layers^14–17^. These studies have reflected that interdependence may induce some new mechanisms that enhance cooperation and fixed the cooperation behavior on the system. And György *et al*. in ref. 18 reviewed how the population structure can modify long-term behavioral patterns in evolutionary games.

In addition to studying how network reciprocity may influence the evolution of cooperation, some researchers have focused on investigating the potential behavior whereby players may adjust their interaction with others based on the gaming results. This is a natural phenomenon since population structure in reality may dynamically change during the game process. A representative method in this subject area is the coevolutionary rule, which was designed and proposed by Zimmermann *et al*. in ref. 19. Even if a large number of studies have contributed to this subject, most works in this area can be divided into those that employ strategy independent rules for connection adaptation^20–22^ and those that take strategies or their performance as factors to influence the population reorganization^23, 24^. Perc *et al*. have also provided a review of this research in ref. 25.

While network reciprocity seems to have preliminarily explained large-scale cooperation in reality, some researchers have practically analyzed the real human game. Their experiment results revealed that humans do not base their strategy decisions on other’s payoffs while playing PDG. In addition, Gracialázaro *et al*. in their experiments have found the existence of a population structure does not seem to have an influence on the global outcome of cooperation^26^. Following these experiments, researchers have also found that cooperation obviously depends on the strategy updating rule. Cimini *et al*. in ref. 27 have analyzed cooperation frequency in a simulation where different strategy updating rules are introduced. They found cooperation frequency assessed under the imitation-based strategy updating rule depends heavily on the population structure, but network reciprocity seems to have little effect on the game dynamics when individuals do not take neighbors’ payoffs into consideration (non-imitative rule). These experiments and extended works seem to have put an end to network reciprocity. However, it remains difficult to conclude that population structure has little effect on promoting cooperation, as different strategy updating rules place different levels of emphasis on different game processes in nature. Evolutionary dynamics based on payoff comparisons are appropriate to model biological evolution, while they may not apply to social or economic contexts^26^. Moreover, Carlos *et al*. has emphasized in their research that their conclusion applies only to human cooperation, and network reciprocity may still be relevant to cooperation in other contexts.

Therefore, even if the relevance between network reciprocity and cooperation in social or economic issues remains controversial, population structure is still essential to cooperation in biological evolution. Moreover, just as group selection indicates that cooperative groups may be more likely to survive in nature than uncooperative ones, cooperation is essential to the survival and evolution of species in nature. A high cooperation level can help species to maintain high competitiveness in nature, which may partially explain why helping family members finally helps the individual itself (Kin selection). Therefore, methods to help optimize the population structure should be important. Even if quite a lot of studies have contributed to the promotion of cooperation in the population, it remains difficult problem^28^. One representative breakthrough that has been made on this subject is the introduction of coevolutionary rules, which has provided a way to help understand the self-reorganizing ability of the population. Even so, little has been done to investigate how a population structure can be constructed or adjusted through man-intervention. And cooperation promotion methods that do not rely on the self-adapt ability of a population may shed light on this problem.

In this paper, we design a variant of evolutionary algorithm to optimize the population structure and thereby enhancing cooperation. Even if much of the preceding research has found a correlation of cooperation and some network properties, naively applying these conclusions to our problems may be quite problematic since these conclusions are mostly obtained based on specific network models and lack generality. Therefore, we employ the cooperation level of population structures as the objective value of our algorithm.

To our knowledge, no appropriate simple approach has been proposed to exactly determine the cooperation level of different population structures. Moreover, the evaluated cooperation level of structures actually fluctuates within a range. Therefore, the evaluation of structures within EA is fuzzy and may interfere with the selection of EA over the elite solutions. In the field of evolutionary computation, researchers also refer to this interference that leads to potential failure in optimization as the “EA cheat”. Apparently, simply employing the canonical evolutionary algorithm (EA) may fail in optimization, even if EA have been applied to many engineering problems^29–36^. Even so, there are two widely accepted methods to reduce the evaluation error of different structures’ cooperation level: (1) Prolong the simulation time of game evolution. (2) Average over independent evaluations. However, the corresponding computation cost cannot be ignored.

To successfully apply EA to the optimization of population structure, we propose a new EA variant named mlEA-C_PD_-SFN to promote cooperation in the Prisoner’s Dilemma Game (PDG). Within mlEA-C_PD_-SFN, a modified local search operator named multilevel evolutionary operator is designed for the purpose of revising the wrong-filtering solution in EA population and exploiting solutions with potential higher cooperation levels. Therefore, we designed a memory structure (restoration list) within the operator to record some reliable solutions for the revision and some rules for the operator to control the search bias.

To test the performance of mlEA-C_PD_-SFN, different types of scale-free structures have been employed, and optimization has been constrained not to change the initial degree distribution. Meanwhile, to verify that the preceding conclusions may not be applied to this problem, we also provide the optimization results of the comparative experiment (EA_cluster_), which optimizes the clustering coefficient of structures. Even if the preceding research concluded that highly clustered scale-free networks enhance cooperation, we still find EA_cluster_ cannot perform as satisfactorily as mlEA-C_PD_-SFN does. Moreover, we also find that the mlEA-C_PD_-SFN can perform well and simultaneously maintain a low computation cost (details in III). Finally, to verify the adaptability of mlEA-C_PD_-SFN subject to different optimization environments, different strategy update rules are employed in our experiments. The simulation results verify the adaptability of mlEA-C_PD_-SFN.

## Results

### Prisoner’s Dilemma Game and Evaluation of Population Structure

Understanding the emergence of cooperation in the context of Darwinian evolution has been a challenge in recent decades. Even if relevant works in this field are almost entirely theoretical, it is quite likely to have broad-reaching implications for the future.

PDG is one of the most commonly used tools to help explain how cooperation endures in nature. In PDG, the defectors receive the highest reward *T* (temptation to defect) when defecting to a cooperator who receives the lowest payoff *S* (sucker value). If both of the players choose the same strategy, they receive a payoff *R* as a reward for cooperation or *P* as punishment for defection. Moreover, *T*, *P*, *S*, *R* follow the rule *T* > *R* > *P* > *S*. As a result, in a single round of PDG, defection is the best strategy no matter what the opponents’ strategies are, even though all players would be better off if they all cooperate.

Population structures provide the basic organization of the game. In ref. 10, players interact only within a limited local neighborhood. When a site *x* is updated, the current occupant and all neighbors around compete to recolonize this site with their offspring. Those offspring keep the same strategy as their parents. The probability of neighbor y succeeding in reproduction is:

1 $${W}_{{s}_{x}\leftarrow {s}_{y}}=\{\begin{array}{c}({P}_{y}-{P}_{x})/(D{d}_{ > }),{P}_{y} > {P}_{x}\\ 0,{P}_{y} < {P}_{x}\end{array}$$

where *d*_>_ = max{*d*_*x*_, *d*_*y*_}, *D* = *T*-*S*, *d*_*i*_ marks the degree of node *i* and *P*_*i*_ marks the payoff of *i*. Therefore, the probability that the focal individual succeeds in reproduction is ${W}_{{s}_{x}\leftarrow {s}_{x}}=\prod _{l}(1-{W}_{{s}_{x}\leftarrow {s}_{y}})$. With probability $1-{W}_{{s}_{x}\leftarrow {s}_{x}}$, an offspring of one neighbor takes over site *x*. The relative probability for the success of neighbor *y* is *W*_*sx*←*sy*_/∑_*l*_ *W*_*sx*←*sy*_, where *l* marks neighbors of *x*. In this paper, the synchronous update method is employed.

Generally, individuals in a population are initially designated as cooperators or defectors with equal probability. And a corresponding cooperation level is obtained through averaging over generations after the equilibrium of a population is reached. Thus, the evaluated cooperation level of structures may naturally fluctuate among independent evaluations.

**Figure 1:**
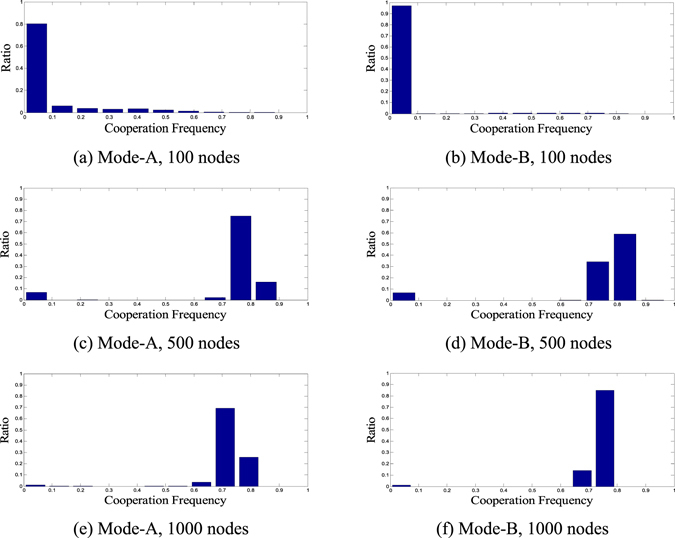
Evaluation distribution of tested population structure (BA network). In this experiment, population structures with different sizes have been tested. It is obvious that the distribution of cooperation frequency obtained under two different evaluation modes is similar (horizontal contrast) and the distribution of cooperation frequency gets broader when size of population gets smaller (vertical contrast).

To illustrate this phenomenon, the distribution of evaluated cooperation level is given in Fig. 1. Each sub-graph in Fig. 1 contains 5000 independent evaluated results of a BA network. Meanwhile, two different evaluation modes have been designed to obtain these simulation results on the same group of population structures: Mode-A and Mode-B. In Mode-A, each equilibrium cooperation level is obtained by averaging 0.1 *N* generations after a transient period of *N* generations. In Mode-B, each equilibrium cooperation level is obtained by averaging *N* generations after a transient period of 10 *N* generations.

Apparently, the independent evaluation results vary within a certain range, which may get wider with the decrease of the tested structure scale. Moreover, dramatically prolonging the simulation time cannot decrease the fluctuation range of evaluation in an obvious way. Meanwhile, the distributions of evaluated cooperation levels obtained under Mode-A and Mode-B are similar, which indicates that Mode-A and Mode-B both provide a necessary transient period for the equilibrium of population.

Therefore, the fluctuation of evaluation may interfere with the selection of EA over the elite solutions. As overlapping may exist between the evaluation distribution of different structures, there naturally exists a certain probability that a worse structure is mistaken by EA as the superior. This phenomenon is termed the “EA cheat” and is a knock down to the algorithm.

**Figure 2:**
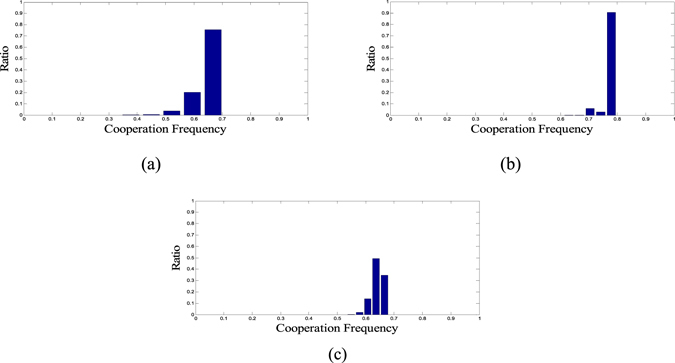
The evaluation distribution obtained under the multi-sampling method. Different sampling numbers have been tested. (**a**) 5 samplings, (**b**) 10 samplings, and (**c**) 20 samplings. On the whole, the multi-sampling method can help reduce the evaluation error of structures. However, different sampling numbers may be necessary toward different evaluation distributions of different structures, which explains why the fluctuation range in Fig. 2(c) is wider than in Fig. 2(b).

To avoid unnecessary computation cost, we employ Mode-A to evaluate the cooperation level of structures in our paper. Moreover, we introduce the multi-sampling method to average over independent evaluations, thereby approaching the ideal mean value of the evaluation distribution. To analyze the effect of this approach, the new evaluation distributions obtained under the multi-sampling method are provided in Fig. 2. On the whole, the multi-sampling method can help reduce the evaluation error of structures. However, different sampling numbers may be necessary toward different evaluation distributions of structures, which explains why the fluctuation range in Fig. 2(c) is wider than that in Fig. 2(b). Therefore, it is difficult to determine an appropriate sampling number, not to mention the fact that this method only decreases the fluctuation range without effectively dealing with the EA cheat (e.g., if the ideal mean value of two evaluation distributions is close enough).

### Multilevel Evolutionary Operator and mlEA-CPD-SFN

Previous simulation results have revealed that the evaluation of structures may fluctuate and interfere with the selection of EA over the elite solutions. Multi-sampling may help reduce evaluation error. But determining an appropriate sampling number will be difficult. Moreover, the corresponding increase in computation cost is unbearable.

Therefore, the reserved (potential) structures should be repeatedly sampled more to ensure reliability of their evaluation results. However, the abandoned (mediocre) structures should be repeat sampled less to decrease computational cost. With this in mind, we propose a local search operator variant named multilevel evolutionary operator (see Methods) to achieve this. And for a timely revision of the wrong-filtering solution caused by the EA cheat, some reliable structures are saved as substitutes and a memory structure (see Methods) is designed. Given these, we further propose a new EA variant named mlEA-CPD-SFN (see Methods) to optimize the structure of scale-free networks for the promotion of cooperation in Prisoner’s Dilemma game.

### Optimizing Clustering Coefficient through EA

Assenza *et al*. have revealed the enhancement of cooperation in highly clustered scale-free networks^12^. Therefore, optimizing the clustering coefficient may help promote the cooperation level of scale-free structures. The objective value in EA_cluster_ is the clustering coefficient of structures, which can be obtained as follows: Suppose neighbors of nodes *i* construct a graph represented by *Γ*_*i*_ and its edge number is |*E*(*Γ*_*i*_)|. Then, the clustering coefficient of node *i* would be *Cluster*_*i*_ = |*E*(*Γ*_*i*_)|/(*d*_*i*_ × (*d*_*i*_ − 1)/2). Therefore, the clustering coefficient of a structure should be *Cluster* = (Σ_*i*_ *Cluster*_*i*_)/*N*.

**Figure 3:**
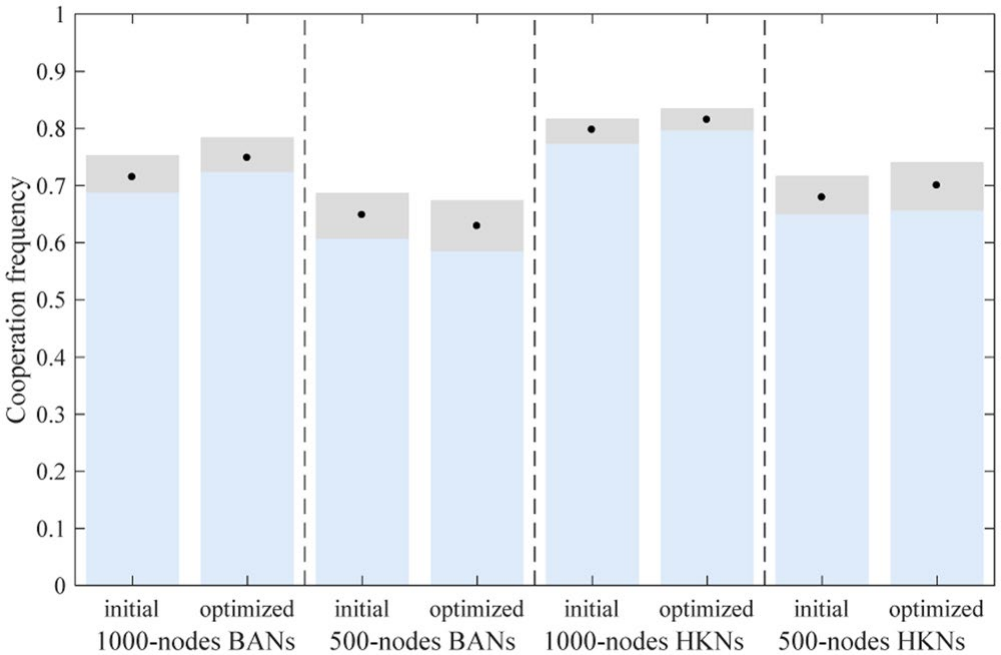
Simulation results of EA_cluster_ in optimizing population structure for the promotion of cooperation in PDG. Each group contains 10 independent structures whose evaluated cooperation level lies within a gray bar (black points mark the mean value). On the whole, naively optimizing the clustering coefficient of a population structure may promote cooperation in PDG, but it is not efficient enough. Moreover, sometimes this method may fail and produce a worse structure, as shown in the optimization of 500-node BANs (the mean value gets smaller).

Two types of scale-free networks are employed to test the performance of EA_cluster_: Barabási Albert networks (BANs)^37^ and Holme and Kim networks (HKNs: *p* = 1). BANs is a type of commonly used scale-free network, while the HKNs is one of its variants and may have higher clustering coefficients in pairs with a higher *p* (*p* ∈ [0,1]) for construction^12^. For fair comparison with mlEA-C_PD_-SFN, canonical local search operator is designed within EA_cluster_, and the maximum generation of EA_cluster_ is set to 120 where evolution of EA_cluster_ has almost converged. The simulation results are given in Fig. 3.

Apparently, optimizing the clustering coefficient of scale-free structures can help promote cooperation in PDG. However, the efficiency of EA_cluster_ is quite limited. Moreover, as EA_cluster_ fails in the optimization of 500-node BANs, we can conclude that naively applying preceding research conclusions to the practical optimization of structures may not perform desirably and sometimes maybe problematic.

### Efficiency of mlEA-C_PD_-SFN in Optimizing Population Structure

In this part, the same structures are employed to test the performance of mlEA-C_PD_-SFN (see Methods). Two types of mlEA-C_PD_-SFN and a hybrid mlEA-C_PD_-SFN are considered:

- Only one level in each pyramid: mlEA-C_PD_-SFN_1_ (shortened to lv1).
- Five levels in each pyramid: mlEA-C_PD_-SFN_5_ (shortened to lv5).
- Only one level in each pyramid but multi-sampling is employed for initial evaluation: mlEA-C_PD_-SFN_1_-M (shortened to lv1-M).

**Figure 4:**
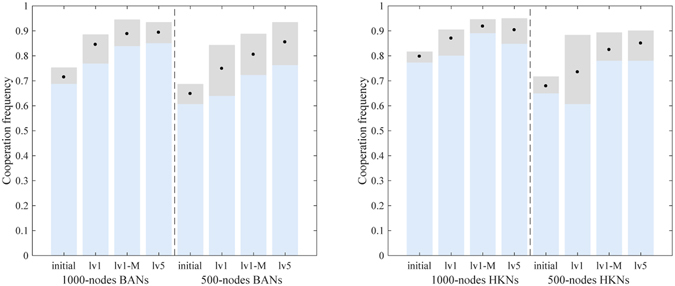
Simulation results of lv1, lv5 and lv1-M in optimizing population structure for the promotion of cooperation in PDG. Each group contains 10 independent structures whose evaluated cooperation level lies within a gray bar (black points mark the mean value). On the whole, these algorithms all efficiently promote cooperation in PDG, and the performance of mlEA-C_PD_-SFN_5_ surpasses those of the others.

To protect the best record in the restoration list, *α* in mlEA-C_PD_-SFN_1_ is set to 0, while *α* = 0.5 in mlEA-C_PD_-SFN_5_. Within mlEA-C_PD_-SFN_1_-M, initial evaluation of structures is obtained by averaging over 5 independent evaluations. The optimization results of these algorithms are given in Fig. 4.

The initial evaluation of structures in mlEA-C_PD_-SFN_1_ and mlEA-C_PD_-SFN_5_ contains only one sampling. Therefore, compared with mlEA-C_PD_-SFN_1_-M, these two mlEA-C_PD_-SFNs in theory are more likely to overestimate or underestimate the cooperation level of structures. As the performance of mlEA-C_PD_-SFN_1_-M surpasses mlEA-C_PD_-SFN_1_, we can conclude that accurate evaluation of structures obviously influences the optimization results. This conclusion also explains why the performance of mlEA-C_PD_-SFN_1_ worsens when optimizing smaller structures. Even if the complexity of a problem synchronously descends with the structure scale, the corresponding evaluation error gets more obvious (refer to Fig. 1). Even so, in our experiments, mlEA-C_PD_-SFN_5_ performs better than mlEA-C_PD_-SFN_1_-M in general. This phenomenon reveals that the level of mlEA-C_PD_-SFN is positive to its performance. The restoration list provides records to maintain the evolving of the EA population subject to the attack of “EA cheat”. With the increase in level, more history records are available, and these records can be saved longer.

**Figure 5:**
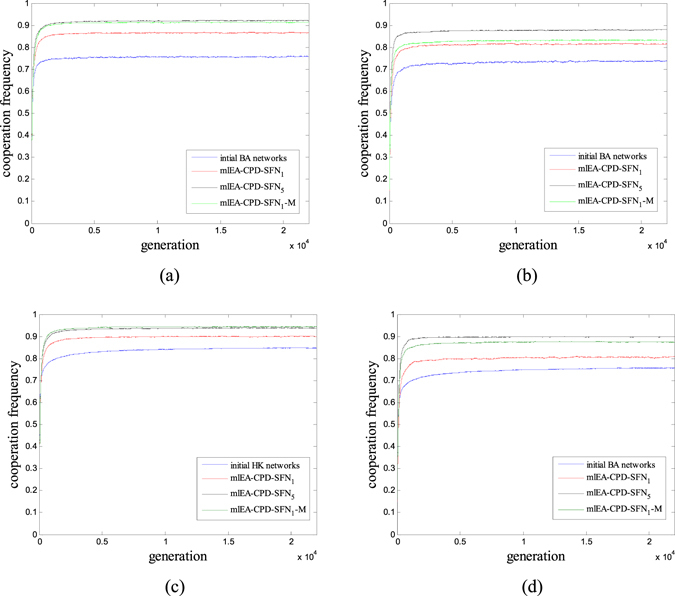
Mean cooperation frequency of structures during the game process. (**a**) 1000-node BANs. (**b**) 500-node BANs. (**c**) 1000-node HKNs. (**d**) 500-node HKNs. Apparently, prolonging simulation time has little influence upon the evaluation results, as cooperation frequency has already converged around 1.1 *N* generation (Mode-A). Moreover, mlEA-C_PD_-SFN_5_ performs best in optimizing population structure and mlEA-C_PD_-SFN_1_-M follows. The optimization effect of these three algorithms is obvious.

The evaluated cooperation level of structures provided in our paper is obtained by averaging over 5000 independent evaluations. Therefore, these simulation results should be reliable. However, further investigation regarding whether prolonging simulation time will influence our results should be undertaken, as those results are obtained under Mode-A with the purpose of saving computation cost. Thus, we prolong the simulation time and track the trend in cooperation during the game process (Fig. 5). Each data point within the simulation results is obtained through averaging over 500 independent runs (50 runs/structure). It is apparent that prolonging simulation time has little influence upon the evaluation results as cooperation frequency has almost converged around 1.1 *N* generation (Mode-A).Therefore, the simulation results we obtain should be credible. In addition, we can conclude that mlEA-C_PD_-SFN performs effectively in optimizing population structure and promoting cooperation in PDG (mlEA-C_PD_-SFN_5_ performs best, and mlEA-C_PD_-SFN_1_-M follows).

We further analyze the optimization characteristic of mlEA-C_PD_-SFN and explore its advantages in optimization. Therefore, we provide the running time and evaluation number of the above three algorithms in Table 1.

**Table 1:** The evaluation number and running time of these three algorithms.

parameter	mlEA-C_PD_-SFN_1_	mlEA-C_PD_-SFN_1_-M	mlEA-C_PD_-SFN_5_
Running time	18893 s	58429 s	20724 s
Evaluation number	143270	454800	135236

Apparently, the computation cost of mlEA-C_PD_-SFN_1_-M surpasses those of the others (almost triple). Thus, the multi-sampling method is paired with a dramatic increase in computation cost. However, mlEA-C_PD_-SFN_*n*_ performs well and simultaneously maintains a low computation cost. This is due to the basic principle of mlEA-C_PD_-SFN: The reserved (potential) structures should be repeatedly sampled more to ensure reliability of their evaluation results. However, the abandoned (mediocre) structures should be repeat sampled less to decrease computational cost. In addition, the structure of the restoration list is irrelevant to the evaluation of structures.

### Adaptability of mlEA-C_PD_-SFN to Different Update Rules

Previous research has concluded that update rules influence the cooperation level of structures^27^. Therefore, investigation of mlEA-C_PD_-SFN’s adaptability to different strategy update rules is necessary. Two common strategy update rules are additionally employed in our experiments:

- Fermi rule: A neighbor (Supposed as *y*) of *x* is chosen randomly. The imitation probability for *y* to learn from *x* is ${W}_{{s}_{x}\leftarrow {s}_{y}}=1/(1+\exp (({P}_{y}-{P}_{x})/k))$, where *P*_*i*_ is the payoff of individual *i* and *k* denotes the amplitude of noise and is set to 0.1.
- Unconditional imitation rule: Each individual *x* imitates the neighbor *y* with the largest payoff, provided *P*_*y*_ > *P*_*x*_.

**Figure 6:**
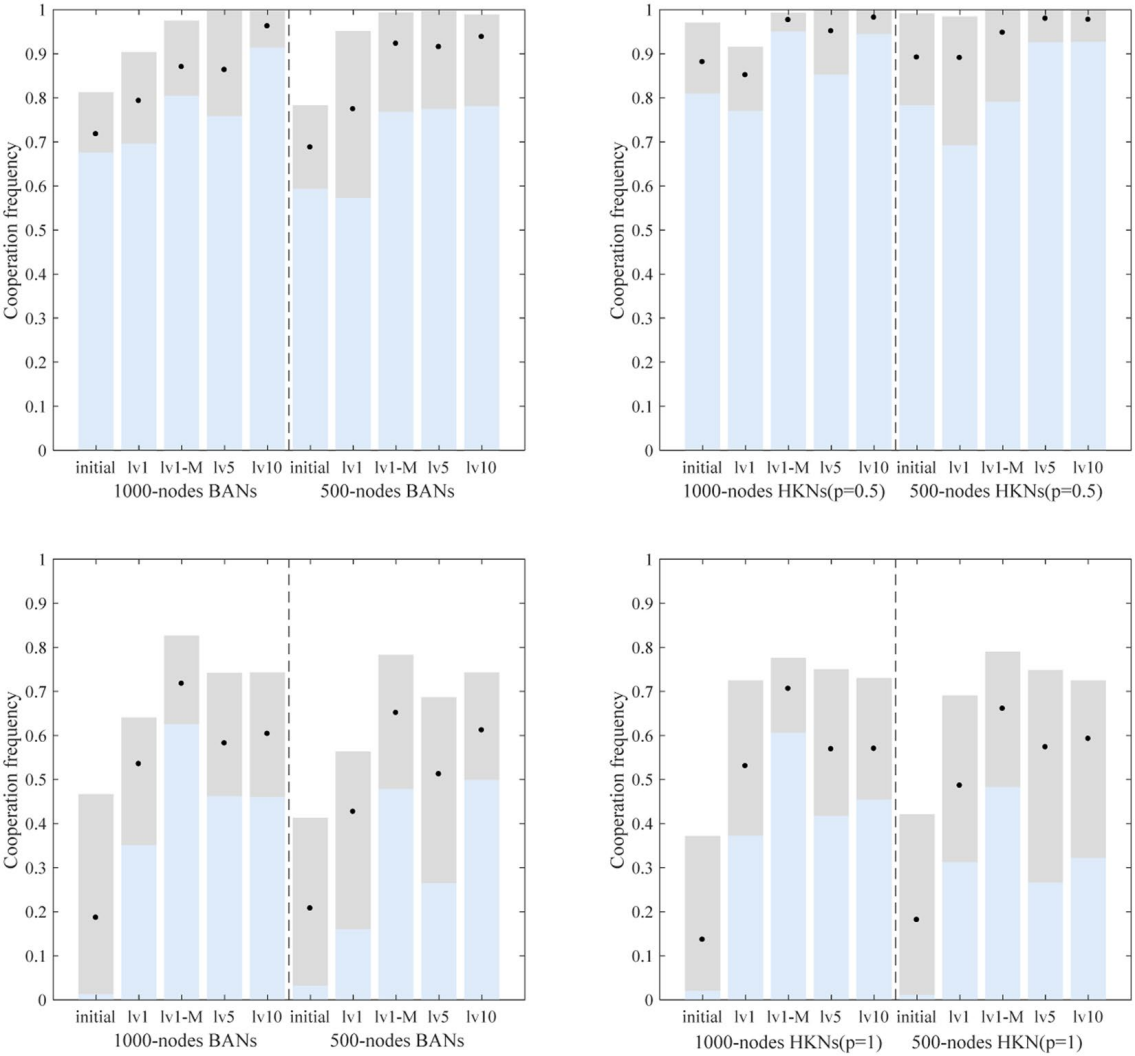
Simulation results of lv1, lv1-M, lv5 and lv10 in optimizing population structure for the promotion of cooperation in PDG. Each group contains 10 independent structures whose evaluated cooperation level lies within a gray bar (black points mark the mean value). The results in the upper (bottom) row are obtained under the Fermi rule (unconditional imitation rule). Overall, these algorithms all effectively promote cooperation in PDG. mlEA-C_PD_-SFN_10_ performs best under the Fermi rule, while mlEA-C_PD_-SFN_1_-M performs best under the unconditional imitation rule.

The configuration of algorithms remains unchanged. As cooperation almost dominates upon HKNs(*p* = 1) under the Fermi rule when the cost to benefit ratio *r* = 0.95, we partially employ HKNs(*p* = 0.5) to test the performance of algorithms. Moreover, performance of mlEA-C_PD_-SFN_10_ (10 levels in each pyramid: lv10) is also provided in Fig. 6. Notably, the simulation time to evaluate the initial and optimized structures in this part is set to 22000 generations, and the equilibrium cooperation level of structures is obtained by averaging the last 2000 generations (like the results in Fig. 5). Therefore, we do not further provide the corresponding mean cooperation frequency of structures during the game process.

Overall, these algorithms all successfully promote cooperation in PDG (Fig. 6). Therefore, we can conclude that mlEA-C_PD_-SFN is adaptable to different strategy updating rules. Moreover, the level of mlEA-C_PD_-SFN apparently influence its efficiency in performance. Finally, we can see mlEA-C_PD_-SFN_1_ apparently fails in some optimization, while mlEA-C_PD_-SFN_1_-M performs well. This phenomenon verifies that the evaluation error may cheat EA and thereby cause the failure of optimization.

Note that unlike other strategy updating rules, the unconditional imitation rule leads to a deterministic dynamic. Therefore, the initialized distribution of strategy plays an important role in the final cooperation frequency, and thereby, the evaluation of structures should be more unstable. This may explain why mlEA-C_PD_-SFN_1_-M performs best under the unconditional imitation rule even if these algorithms all perform effectively.

## Discussion

Cooperation is essential in many aspects of life. In biology, the prisoner’s dilemma game (PDG) has long been used to help explain how cooperation endures in nature. As cooperation is highly relevant to the competitiveness of groups in nature, understanding cooperation in PDG and proposing methods of promotion should have significant implications. To our knowledge, although many well-known mechanisms have provided ways to understand the self-reorganizing ability of a population toward an optimal situation for cooperation, little has been done to investigate how to construct or adjust the population structure with man-intervention, even if the preceding research recognizes its importance. Therefore, cooperation optimization methods that do not rely on the self-regulation mechanism of a population may shed light on this problem.

The contributions of this paper are summarized as follows: (1) We propose a new EA variant named mlEA-C_PD_-SFN to optimizes the structure of scale-free networks for the promotion of cooperation in the Prisoner’s Dilemma game without changing the initial structures’ degree distribution. (2) We reveal that evaluation error of population structures may cause the “EA cheat” and canonical evolutionary algorithm (EA) may fail in optimization. (3) Different types of scale-free structures and updating rules have been applied to verify the performance of mlEA-C_PD_-SFN. (4) We provide the optimization results of the comparative experiment (EA_cluster_) and reveal that naively applying preceding research conclusions to the practical optimization of structures may not perform desirably and sometimes maybe problematic. (5) The experimental results show that mlEA-C_PD_-SFN can perform well in various situations and simultaneously maintain a low computation cost.

We hope mlEA-C_PD_-SFN may help promote the structure of species in nature and that more general properties that enhance cooperation can be learned from the output structures.

## Methods

### Restoration List

**Figure 7:**
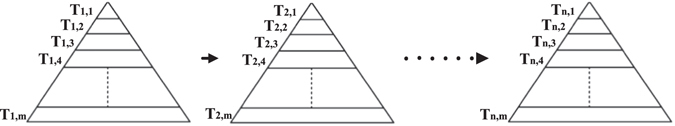
Restoration list.

Even if the “EA cheat” may lead to some wrong-filtering solution in the EA population, timely revision still may save EA from failure in optimization. Therefore, a hierarchical memory structure (given in Fig. 7) is designed to backup some reliable solutions in case of need. There are 2*Ω* pyramid-like sub-lists in the restoration list, and each contains some vacant spaces for records. Notably, records in the upper pyramid are prior to those in the bottom.

*L*_*n*,*m*_ marks the *m*th record of the *n*th pyramid, and *L*_*n*_ marks the *n*th pyramid. *L*_*n*_ is responsible for *n*th solution in the EA population. The reason to separate the memory structure into numbers of parts is: (1) Maintain the diversity of the EA population and to avoid premature convergence of algorithm. (2) Maintain the parallelism of the EA. (3) Enable communication among pyramids with the help of computing characteristic of EAs.

### Basic Strategies for Multilevel Evolutionary Operator

The basic rules for multilevel evolutionary operator are: (1) Some supplementary rules for the restoration list. (2) Rules to compare structures with different sampling numbers. Note that *sum* marks the sum of evaluation results and *num* marks the number of evaluations (sampling number); e.g.: if a structure has been evaluated twice, *num* = 2 and *sum* is the total of the evaluation results. Therefore *I*_*i*_ = {*G*_*i*_, *sum*_*Gi*_, *num*_*Gi*_} marks the *i*th solution in the EA population. *G* = (*V*, *E*) is used to represent a population structure (graph), where *V* = {*v*_1_, *v*_2_, …, *v*_*N*_} is the set of nodes and *E* = {*e*_*ij*_ | *i*, *j* ∈ *V* and *i* ≠ *j*} is the set of edges in the structure.

(1) Four rules are designed for the restoration list: *insertion rule*, *mutation rule*, *sorting rule*, and *information update rule*.

- The *insertion rule*: Suppose we attempt to backup the *i*th solution to the restoration list. If there are vacant spaces in ***L***_*i*_, this solution is inserted directly at the bottom of ***L***_*i*_. Otherwise, this solution should clear out some old records with smaller *avg* = *sum*/*num* and *num* first. If no vacant space is ready, this solution will not be backed up.
- The *mutation rule*: Suppose *i*th solution has a larger *avg* than *L*_*i*,*m*_. Even if the insertion rule is unsatisfied, it may still take the place of *L*_*i*,*m*_ with probability *α* ^*max-m*^ (*max* is the number of the vacant space in *L*_*i*_).
- The *sort rule*: Solutions in pyramids are prioritized in terms of their *avg*, and *sort_list*(*i*) marks the sorting operation on the *i*th pyramid.
- The *information update rule*: The backup should be updated synchronously if the *sum* or *num* of its ontology is updated and *record_synchronize*(*L*_*i*,*m*_, *sum*, *num*) is used to denote the update operation of *L*_*i*,*m*_.

(2) Two comparison strategies are designed to compare structures with different sampling numbers. **Strategy** 1 is used to compare solutions in the EA population and **strategy** 2 is used to compare the solutions in the EA population and restoration list.

**Strategy 1** (*I*_*i*_ and *I*_*j*_): if *avg*_*Gi*_ > *avg*_*Gj*_, *I*_*i*_ is better than *I*_*j*_.

**Strategy 2** (*I*_*i*_ and *I*_*j*_ (record)): We suppose *G*_*i*_’ is a variant from *G*_*i*_. (i) Only when *avg*_*Gi*_ < *avg*_*Gi’*_ < *avg*_*Gj*_, *I*_*j*_ is considered more optimal than *I*_*i*_. (ii) Only when *avg*_*Gi’*_ > *avg*_*Gi*_ > *avg*_*Gj*_, *I*_*i*_ is considered more optimal than *I*_*j*_. Details of how *G*_*i*_’ is produced are given below.

### Multilevel Evolution Operator: A Variant from the Local Search Operator

**Figure 8:**
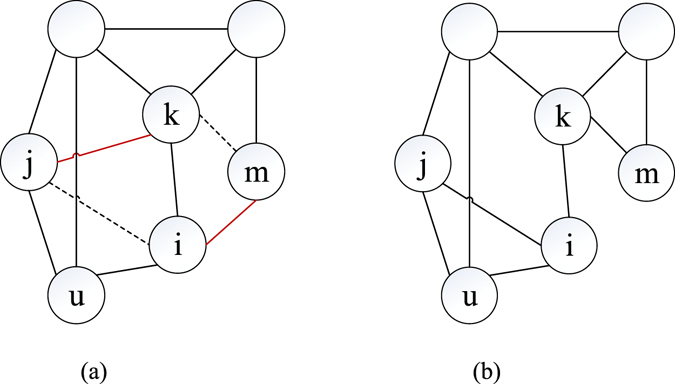
Structure adjusted before and after edge switching. (**a**) Initial structure *G*_*i*_. (**b**) Structure adjusted (*G*_*i*_’). Remove *e*_*jk*_ and *e*_*im*_ from structure *G*_*i*_ in (**a**); then, *e*_*ji*_ and *e*_*km*_ are added, thereby gaining a new structure *G*_*i*_’ in (**b**).

Local search operator is a widely employed method to improve the exploitation of EA through continuous fine-tuning of solutions. In our paper, we employed an edge switching process (in Fig. 8) to complete the fine-tune of the current solution *G*_*i*_ and obtain *G*_*i*_’: (a) A node *u* (*d*_*u*_ ≥ 2) with its two neighbors *i* and *j* (*d*_*i*_, *d*_*j*_ ≥ 2) are selected. (b) Edges *e*_*jk*_ and *e*_*im*_ (*u* ≠ *i* ≠ *j* ≠ *k* ≠ *m*) are selected. (c) Remove *e*_*jk*_, *e*_*im*_ and add *e*_*ji*_, *e*_*km*_ (*e*_*ji*_, *e*_*km*_ ∉ *G*_*i*_). *edge_switch*(*G*_*i*_, *v*_*u*_, *v*_*j*_, *v*_*i*_, *v*_*k*_, *v*_*m*_) marks this edge switching operation (step c), and *node_select*(*v*_*u*_, *v*_*j*_) marks the node selecting process (step a-b).

Canonical local search operator only compares and selects between the initial and adjusted solutions in terms of their evaluation results. However, the records in the restoration list are also considered by the multilevel evolutionary operator (Suppose *I*_*i*_, *I*_*i*_’ mark the initial solution and adjusted solution, while *L*_*i*,0_ is the top level record in the *i*th pyramid):

- If *avg*_*Gi*_ > *avg*_*Gi*’_: (a) *I*_*i*_’ is abandoned, and *I*_*i*_ is reserved. (b) Evaluate the structure of *I*_*i*_ once more, and update *sum* and *num*. (c) Synchronize information if its backup exists.
- If *avg*_*Gi*’_ > *avg*_*Gi*_ > *avg*_*Li*_: (a) Backup *I*_*i*_. (b) *I*_*i*_ is replaced by *I*_*i*_’. (c) Sort records in *L*_*i*_.
- If *avg*_*Li*_ > *avg*_*Gi*’_ > *avg*_*Gi*_: Replace *I*_*i*_ with *L*_*i*,0_ and *restoration*(*i*) denotes this operation.
- For other situations, the multilevel evolution operator replaces *I*_*i*_ with *I*_*i*_’.

**Algorithm 1** Provide details of the multi-level evolutionary operator and *β* marks the probability scale.

**Table:** 

**Algorithm 1: Multilevel Evolutionary Operator**
**Input**: *I*_*k*_: *k*th solution in EA population, *avg*_*Gk*_: Evaluation of *G*_*k*_; *β*: probability scale.
**Output**: *Ĩ*_*k*_’: modified solution from *I*_*k*_. *avg*_*Ĩk*’_: Evaluation of *Ĩ*_*k*_’;
Step 1: Initialization, see ***algorithm 2*** for more information.
Step 2: Local search based on the hill-climbing, iterates over every edge of nodes.
(a) Edge switching (see ***algorithm 3*** for more information).
(b) If the adjusted solution is unreasonable, conduct ***algorithm 4***.
(c) If the adjusted solution is accepted, operates according to the specific situations. See ***algorithm 5*** for more information.
(d) If the loop is over go to step 3, else go to the next round.
Step 3: Output current *I*_*k*_ and *avg*_*GK*_ as *Ĩ*_*k*_’ and *avg*_*Ĩk*’_;

**Table:** 

**Algorithm 2: Initialization**
1: *Flag*_*List*_←*false*;//*Flag*_*List*_ marks whether the current structure has already been backed up;
2: *sum*_*Gk*_← *avg*_*Gk*_,
3: *num*_*Gk*_←1;

**Table:** 

**Algorithm 3: Edge switching adjustment**
1: ***if***(*U*(0,1) < *β*×*d*_*i*_/∑_*i*_ *d*_*i*_) ***then***
2: *[Is_Nodes_Found*, *v*_*i*_, *v*_*a*_, *v*_*b*_, *v*_*c*_, *v*_*d*_ *]*←*node_select*(*v*_*i*_, *v*_*a*_);
3: ***if***(*Is_Nodes_Found* = *false*) ***then***
4: ***continue***;
5: ***else***
6: *G*_*k*_‘ ← *edge_switch*(*G*_*k*_, *v*_*i*_, *v*_*a*_, *v*_*b*_, *v*_*c*_, *v*_*d*_);
7: ***else***
8: ***continue***;
9: ***end if*** **;**
10: avg_*Gk*_‘ ← *C*(*G*_*k*_‘);//*C*(*G*_*k*_‘) evaluate cooperation level of *G*_*k*_‘ once;

**Table:** 

**Algorithm 4: Operation when edge switching adjustment is rejected**
1: ***if***(*G*_*k*_‘ is not connected or avg_*Gk*‘_ < avg_*Gk*_) ***then***
2: *num*_*Gk*_←*num*_*Gk*_ + 1;
3: *sum*_*Gk*_←*sum*_*Gk*_ + *C*(*G*_*k*_);
4: *avg*_*Gk*_←*sum*_*Gk*_/*num*_*Gk*_;
5: ***if***(*Flag*_*List*_) ***then***
6: *record_synchronize* (*i*, *sum*, *num*)
7: ***end if***;
8: ***end if***;

**Table:** 

**Algorithm 5: Operation when edge switching adjustment is accepted**
//*avg*_*Gk’*_ > *avg*_*Gk*_: adjustment accepted;
1: ***if***(*avg*_*Gk*_ > *avg*_*Li*_) ***then***//*avg*_*Gi*’_ > *avg*_*Gi*_ > *avg*_*Li*_;
2: Attempt to back-up *G*_*k*_ in *L*_*k*_;
3: *sort_list*(*k*);
4: *avg*_*Gk*_← *avg*_*Gk’*_;
5: *num*_*Gk*_←1;
6: *sum*_*Gk*_← *avg*_*Gk’*_;
7: *Flag*_*List*_←*false*;
8: *G*_*k*_←*G*_*k*_‘;
9: ***elseif***(*avg*_*Li*_ > *avg*_*Gk’*_) ***then*** **/**/*avg*_*Li*_ > *avg*_*Gi*’_ > *avg*_*Gi*_
10: *restoration*(*k*);
11: *is_list_taken*←*true*;
12: ***else***
13: *avg*_*Gk*_← *avg*_*Gk’*_;
14: *num*_*Gk*_←1;
15: *sum*_*Gk*_← *avg*_*Gk’*_;
16: *Flag*_*List*_←*false*;
17: *G*_*k*_←*G*_*k*_‘;
18: ***end if***;

### Implementation of mlEA-C_PD_-SFN

**Figure 9:**
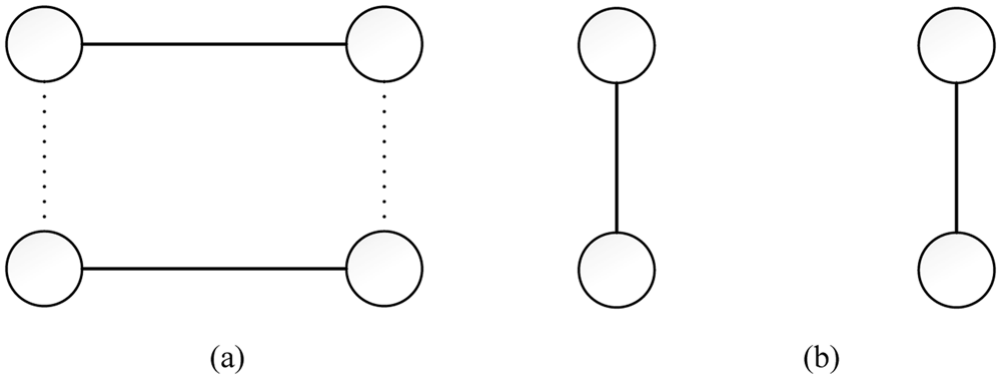
Structures before and after being adjusted by simple edge swapping. (**a**) Initial structure. (**b**) Structure adjusted. The solid lines mark the current existing edges, while the dotted lines mark the targeted edges to be constructed.

Given a targeted population structure for optimization, we first generate an EA population {*G*_1_, *G*_2_, …, *G*_Ω_} through a simple edge swapping operation (Fig. 9). Crossover operators of mlEA-C_PD_-SFN is similar to the crossover operation in^33^ **Algorithm 6** provides the details of mlEA-C_PD_-SFN.

**Table:** 

**Algorithm 6: mlEA-C**_**PD**_ **-SFN**
**Input**: *G*_0_: Initial scale-free structure. *GS*: Population size. *P*_*c*_: Crossover rate. *max*: Levels in pyramids. *gen_max*: Iteration number. *β*: probability scale.
**Output**: G*: The optimized population structure.
Step 1: Initialize population through *simple_edge_swapping*(*G*0) and evaluate their cooperation level;
Step 2: EA population reproduce (Crossover operator). Select parents through roulette selection.
_Step 3: Mutation (*simple_edge_swapping*) to the offspring_ and evaluate their cooperation level.
Step 4: Multilevel evolutionary operator performs upon both the parent and the offspring.
Step 5: If the iteration generation satisfies, output the best solution in EA population. Or else, select the best *GS* solutions in the EA population for the next generation and go to step 2.

Some parameters in experiments are fixed as: *P* = 0, *T* = 1 + *r*, *S* = 0, *R* = 1, *β* = 20, *r* = 0.95 (details of cost to benefit ratio *r* in ref. 10), *GS* = 6 and *gen_max* = 30. Notably, the cooperation level of structures provided in **Results** is obtained by averaging over 5000 independent evaluations.
